# Associations of decayed teeth and localized periodontitis with mental stress in young adults: CHIEF oral health study

**DOI:** 10.1038/s41598-022-23958-4

**Published:** 2022-11-09

**Authors:** Kun-Zhe Tsai, Sung-Chiao Tsai, Ko-Huan Lin, Yun-Chen Chang, Yen-Po Lin, Gen-Min Lin

**Affiliations:** 1 Department of Stomatology of Periodontology, Mackay Memorial Hospital, New Taipei City, Taiwan; 2 Department of Medicine, Hualien Armed Forces General Hospital, No. 163, Jiali Rd., Xincheng Township, Hualien, 97144 Taiwan; 3 Departments of Dentistry, Tri-Service General Hospital, National Defense Medical Center, Taipei, Taiwan; 4 Department of Psychiatry, Taipei Veterans General Hospital Yuli Branch, Hualien, Taiwan; 5 School of Nursing and Graduate Institute of Nursing, China Medical University, Taichung, 406 Taiwan; 6 Nursing Department, China Medical University Hospital, Taichung, Taiwan; 7 Department of Critical Care Medicine, Taipei Tzu-Chi Hospital, New Taipei City, Taiwan; 8 Department of Internal Medicine, Tri-Service General Hospital, National Defense Medical Center, Taipei, Taiwan

**Keywords:** Psychology, Diseases, Risk factors

## Abstract

The associations of mental stress with decayed teeth in children and periodontitis in old-aged adults have been described. However, the associations for young adults were not clear. This study aimed to examine the associations of decayed teeth and localized periodontitis with mental stress in young adults. This study included 334 military recruiters, aged 19–45 years in Taiwan. Mental stress was assessed by the brief symptom rating scale-5 (BSRS-5), including five domains: anxiety, depression, hostility, interpersonal sensitivity and insomnia (maximum score of 20). Those with symptomatic mental stress were defined as having BSRS-5 > 5 (n = 34). Multiple linear and logistic regression models were used to determine the associations of decayed tooth numbers and periodontitis with BSRS-5, with adjustments for age, sex, education level, physical activity, body weight category and smoking status. The BSRS-5 was positively correlated with decayed tooth numbers [β: 0.26 (95% confidence interval: 0.01–0.52)]. Those who had more than two decayed teeth [odds ratio: 3.59 (1.52–8.46)] had a higher risk of symptomatic mental stress. In contrast, the correlation between BSRS-5 and localized severer periodontitis was null. Our study recommended that decayed teeth instead of localized periodontitis, was a risk factor for mental stress in young adults.

## Introduction

Psychological stress is often present in military personnel, who have to take multiple deployments, heavy training, and responsibility^1^. The prevalence of symptomatic mental stress varies from 2.0 to 37.4% in military personnel worldwide^1–4^. Under stress, organisms might present corresponding physical responses in order to adapt to the situation; however, metnal stress would have detrimental effects when it remains constant^5,6^. In the oral cavity, recurrent aphthous stomatitis^7^, temporomandibular disorder^8^, myofascial pain^9^, poor oral hygiene^10^, periodontal disease^11^ and dental caries^12^ have been reported to be related to mental stress.

Dental caries, otherwise known as tooth decay, is the most common, chronic, noncommunicable, preventable oral disease worldwide^13^. The World Health Organization (WHO) reported that 95% of the world population has suffered from dental caries^14^. Dental caries form through a complex interaction over time between acid-producing bacteria and fermentable carbohydrates and many host factors, such as teeth and saliva^15^. Many studies indicated a positive association between the presence of decayed teeth and salivary cortisol levels, a potential mental stress biomarker^16^, in primary and mixed dentition^12^ but not in permanent dentition. In addition, it is inconvenient to detect salivary cortisol in the dental clinical environment.

Periodontal disease is also a common disease in the oral cavity^17^. Past studies have revealed that there is a positive correlation between mental stress and generalized periodontitis in middle-aged and elderly individuals^11,18^. However, the association with localized periodontitis in young adults has rarely been explored. Thus, we used the Brief Symptom Rating Scale-5 (BSRS-5), a short questionnaire with five questions, to inspect the associations of mental stress with the number of decayed teeth and localized periodontitis in young military personnel.

## Methods

### Study population

The present study used a historical cohort of 334 military personnel, averaged 32.6 years of age from the cardiorespiratory fitness and health in eastern armed forces (CHIEF) oral health study^19^ performed in Taiwan during 2018. Subjects were divided into two groups (normal and those with psychological stress) according to the BSRS-5 score^20–23^. All participants self-reported a questionnaire for education level, exercise activity, smoking status and psychological status assessments. Participants also received an annual military health examination, including oral health and physical and laboratory examinations. This study design and protocol were reviewed and approved by the human ethics board of the Mennonite Christian Hospital (No. 16-05-008) in Hualien, Taiwan and written informed consent was obtained from all subjects. All methods were performed in accordance with relevant guidelines and regulations.

### Clinical and demographic measures

For each participant, body height and body weight were assessed in a standing position. Body mass index (BMI) was defined as the ratio of body weight (kg) to the square of height (m^2^). Since participants were all Taiwanese, overweight and obesity were respectively defined as BMI: 24.0–27.4 kg/m^2^ and ≥ 27.5 kg/m^2^ using the Taiwanese criteria^24^. Measurement of waist circumference was taken at the midpoint between the highest point of the iliac crests and the lowest point of palpable ribs. Hemodynamic parameters of each subject, such as systolic blood pressure (SBP) and diastolic blood pressure (DBP), were measured after resting for at least 25 min and taken once from the right arm in a sitting position with an automated FT-201 BP device (Parama-Tech Co Ltd, Fukuoka, Japan). Routine blood tests, including leukocyte count, fasting glucose, total cholesterol, high-density lipoprotein, low-density lipoprotein and triglycerides, were measured using an automated hematology analyzer^25,26^ (Olympus AU640 autoanalyzer, Olympus, Kobe, Japan). All blood samples of participants were collected after an overnight 12-h fast at the same blood drawing station.

### Oral health measures

The periodontal charting consisted of probing pocket depth (PPD), which was measured to the closest millimeter with a periodontal probe (Hu-friedy color-coded single-end probe, USA) at 6 sites (mesiobuccal, midbuccal, distobuccal, mesiolingual/palatal, midlingual/palatal and distolingual/palatal) per tooth^27^, except third molar and impacted teeth. Clinical attachment loss (CAL) was calculated as a sum of PPD and cemento-enamel junction scores. In addition, the oral examination also included furcation involvement and tooth mobility. Furcation involvement was classified into three degrees (1, 2 and 3) according to horizontal attachment loss from furcation entrance. Tooth mobility was defined as grades 0, 1, 2, 3, using the Miller classification. In addition, well oral hygiene status defined as tooth brushing ≥ 2 times per day was also reported for each participant.

Dental caries was assessed by recording the caries index, DMFT (number of decayed, missing and filled teeth), according to the criteria recommended by the WHO^28^. Impacted teeth and third molars were excluded from this study. The selected subjects in the present study with dental caries had a history of pain or infection. Comprehensive oral treatment was given to all subjects after recording the baseline DMFT scores. The severity of localized periodontitis was classified based on the 2017 world workshop of both the American Academy of Periodontology and the European Federation of Periodontology^29^. For all participants, the extent of periodontitis was defined as localized (< 30% of teeth involved)^27^. Localized stage II and III periodontitis were defined as localized more severe periodontitis in this study. Stage II periodontitis was defined if either one of the criteria fulfilled the following: (1) interdental CAL: 3–4 mm at the side of greatest loss; (2) radiographic bone loss: 15–33%; (3) no tooth loss due to periodontitis; or (4) maximum PPD ≤ 5 mm or horizontal bone loss. Stage III periodontitis was diagnosed if either one of the following criteria were met: (1) interdental CAL ≥ 5 mm at the side of greatest loss; (2) radiographic bone loss extending to the mid-third of the root and beyond; (3) tooth loss due to periodontitis; (4) PPD ≥ 6 mm, vertical bone loss ≥ 3 mm, class II/III furcation involvement, or moderate ridge defect. Within one month, a follow-up check-up and treatment for the oral pathologies of each participant was performed by other dentists in the Outpatient Department, and the inter-observer agreement (kappa coefficient) for the periodontitis stage was estimated as 90.6%^30^.

### Mental stress measurements

The BSRS-5 score has been used to evaluate the severity of recent (within weeks) mental stress of military personnel in Taiwan for several years. The BSRS-5 score is composed of five domains, including feeling blue (depression), feeling tense (anxiety), feeling easily annoyed or irritated (hostility), feeling inferior to others (interpersonal sensitivity), and trouble falling asleep (insomnia). The scoring of each domain ranges from 0 (none) to 4 (extremely) in severity^20^, and thus the BSRS-5 score ranges from 0 (none) to 20 (extremely). Participants with a BSRS-5 score > 5 were defined as having symptomatic mental stress, and participants with a BSRS-5 score ≤ 5 were defined as the normal mental group^31,32^. Internal consistency (Cronbach’s alpha) coefficients of the BSRS-5 score have been reported to range from 0.77 to 0.90. The test–retest reliability coefficient was 0.82^20^.

### Statistical analysis

**Figure 1:**
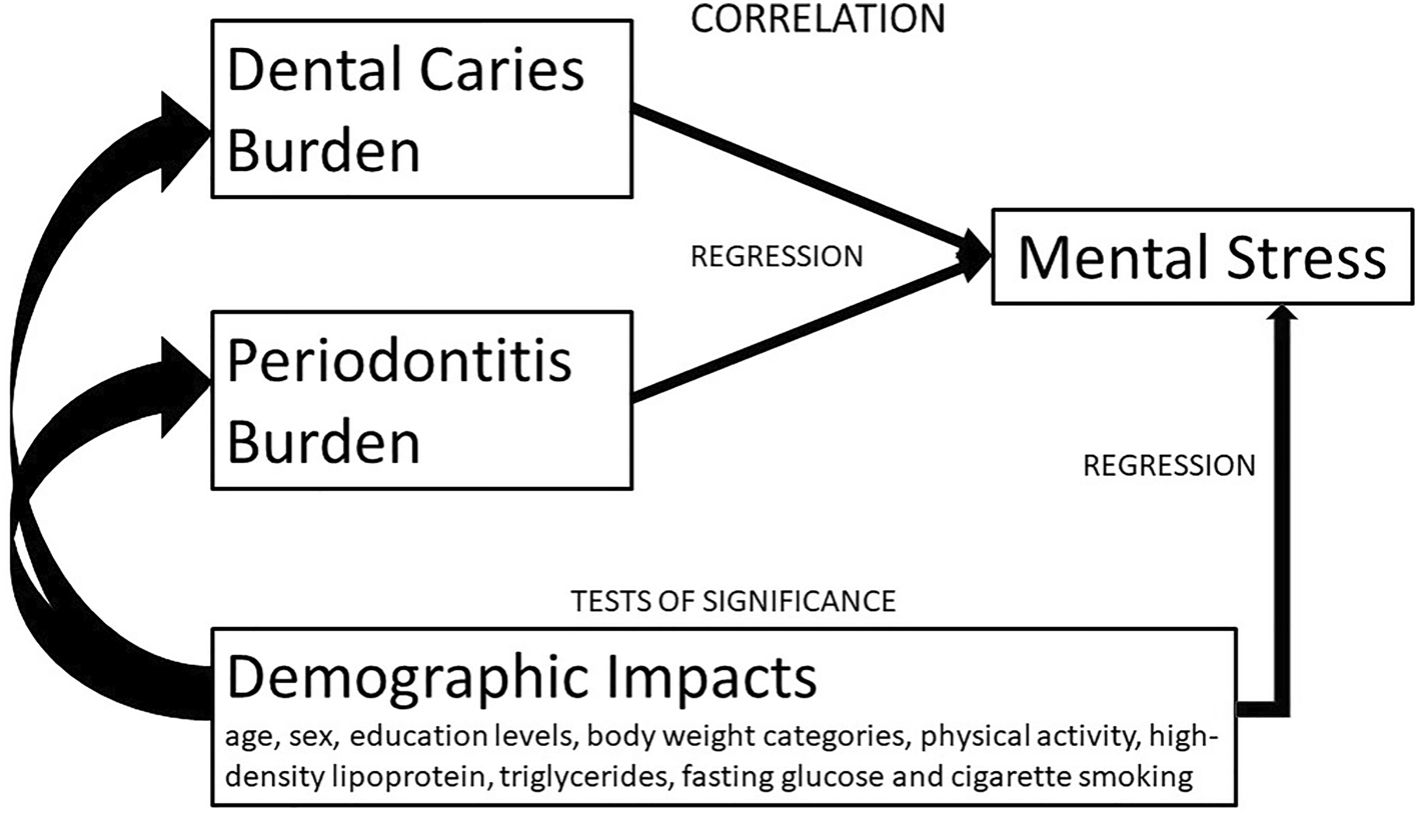
The theoretical framework for the present study.

The theoretical framework of this cross-sectional study was shown in Fig. 1. The variables were expressed as the mean ± standard deviation (SD) for continuous data, and numbers (%) for categorical data. Continuous variables were compared by one-way ANOVA, and categorical variables were compared by the chi-square test. Multivariable linear regression analysis was used to separately determine the β value and their 95% confidence intervals (CI) of 6 exposure variables, i.e., the BSRS-5 score and related 5 central domains, anxiety, depression, hostility, interpersonal sensitivity and insomnia, with the outcome variable of decayed tooth numbers. Multivariable logistic regression analysis was also used to separately determine the odds ratio (OR) and the 95% CI of 2 exposure variables, i.e., more than two decayed teeth and localized severer periodontitis with the outcome variable of symptomatic mental stress. The covariates that were related to both dental diseases and mental stress were selected on the basis of previous studies, and stepwise adjusted in three models for decayed tooth numbers and localized severer periodontitis. In Model 1, age, sex and education level were adjusted. In Model 2, body weight categories and physical activity were additionally adjusted. In Model 3, smoking status and the metabolic biomarkers which had a *p*-value < 0.20 between participants with and without symptomatic mental stress, i.e., plasma high-density lipoprotein, triglycerides and fasting glucose were chosen for adjustments. A sensitivity test for unmeasured confounding was also performed using the method by Lin et al.^33^. A value of *p* < 0.05 was considered significant. SPSS statistical software was used for all statistical analyses (IBM SPSS Statistics for Windows, Version 25.0. International Business Machines Corporation, Armonk, NY, USA).

## Results

**Figure 2:**
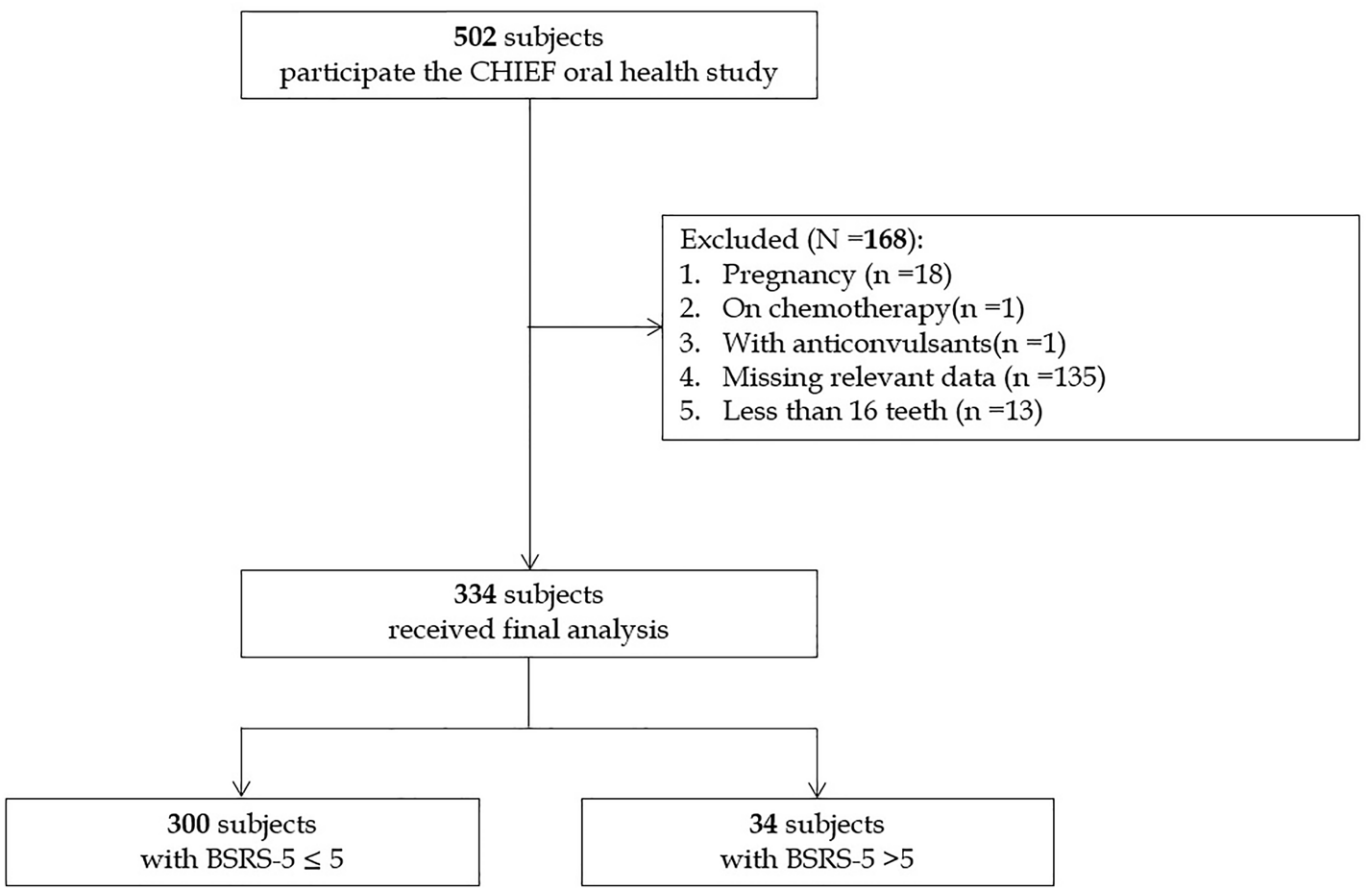
Flow diagram to select the military participants for analysis.

Table 1 shows the clinical characteristics of participants with normal (N = 300) and symptomatic mental stress (N = 34). The flow diagram for the selection of participants is shown in Fig. 2. Those with symptomatic mental stress had a lower serum HDL-C level, lower physical activity and a greater BSRS-5 score. There were no differences in prevalence of oral hygiene status, decayed tooth numbers, the median of the DMFT index, and Stage II/III periodontitis.

**Table 1:** Clinical characteristics of the normal and the symptomatic mental stress groups (N = 334).

Clinical characteristics	Normal BSRS-5 ≤ 5 (N = 300)	Mental stress BSRS-5 > 5 (N = 34)	*p*-value
BSRS-5 scores	1.36 ± 1.76	7.53 ± 1.56	< 0.001
Sex			
Male	270 [90.0]	32 [94.1]	0.43
Female	30 [10.0]	2 [5.9]	
Age (years)	32.74 ± 4.86	31.47 ± 4.00	0.14
Education level			
Senior high school	17 [5.7]	2 [5.9]	0.62
College/University degree	275 [91.7]	32 [94.1]	
Postgraduate degree	8 [2.7]	0 [0.0]	
Tooth brushing ≥ 2 times per day	272 [90.7]	30 [88.2]	0.64
Physical activity			
Never or occasionally	54 [18.0]	8 [23.5]	< 0.001
1–2 times per week	120 [40.0]	24 [70.6]	
≥ 3–5 times per week	126 [42.0]	2 [5.9]	
Active cigarette smoker	102 [34.0]	10 [29.4]	0.59
Systolic blood pressure (mmHg)	121.14 ± 12.23	122.50 ± 11.79	0.53
Diastolic blood pressure (mmHg)	73.47 ± 9.77	74.47 ± 10.01	0.57
Body mass index (kg/m^2^)	25.80 ± 3.21	25.30 ± 2.60	0.38
Obesity	93 [32.3]	9 [26.5]	0.47
Overweight	110 [36.7]	11 [32.4]	
Normal weight	93 [31.0]	14 [41.2]	
Waist circumference (cm)	85.90 ± 8.63	85.04 ± 5.96	0.57
Blood test			
Total cholesterol (mg/dL)	181.20 ± 34.19	176.88 ± 36.82	0.48
HDL-C (mg/dL)	48.93 ± 10.56	44.35 ± 9.41	0.01
LDL-C (mg/dL)	109.17 ± 32.37	105.71 ± 31.68	0.55
Serum triglycerides (mg/dL)	132.61 ± 94.54	154.79 ± 99.63	0.19
Fasting glucose (mg/dL)	93.02 ± 15.06	98.26 ± 23.41	0.07
Leucocyte count (10^9^/L)	6.92 ± 1.60	7.04 ± 1.45	0.67
*DMFT index [median (25th, 75th)]	4 (1, 7)	3 (1.75, 6.50)	0.10
Range (min–max)	(0–16)	(0–14)	
Decayed teeth (numbers)	0.54 ± 1.33	0.93 ± 1.08	0.12
Missing teeth (numbers)	1.27 ± 1.58	1.13 ± 0.97	0.65
Filled teeth (numbers)	5.32 ± 3.84	4.67 ± 3.92	0.38
Localized stage II and III periodontitis	84 [28.0]	10 [24.9]	0.86

Continuous variables are expressed as mean ± SD (standard deviation), and categorical variables as N [%].

*BSRS-5* brief symptom rating scale-5, *DMFT* decayed, missing and filled teeth numbers, *HDL-C* high-density lipoprotein cholesterol, *LDL-C* low-density lipoprotein cholesterol.

*DMFT index was presented as median (25%, 75%) for the distribution skewed to left.

Table 2 reveals the results of multivariable linear regressions for decayed tooth numbers with BSRS-5 score and its components in Models 1–3. Greater decayed tooth numbers were positively correlated with greater BSRS-5 scores [β: 0.26 (95% CI: 0.01–0.52)] and its components of anxiety [β: 0.06 (95% CI: 0.01–0.12)], depression [β: 0.06 (95% CI: 0.01–0.12)] and interpersonal sensitivity [β: 0.07 (95% CI: 0.01–0.13)] in Model 3 and in the other 2 models. Although there was a positive association of decayed tooth numbers with insomnia in Models 1 and 2, the associations became borderline in Model 3. There were no associations of decayed tooth numbers with hostility in Models 1–3.

**Table 2:** Multivariable liner regression analysis for decayed tooth numbers with BSRS-5 score.

	Model 1			Model 2			Model 3		
	β	95% CI	*p*-value	β	95% CI	*p*-value	β	95% CI	*p*-value
BSRS-5 scores	0.30	0.06–0.54	0.01	0.27	0.03–0.51	0.02	0.26	0.01–0.52	0.04
Anxiety	0.07	0.02–0.13	0.006	0.07	0.01–0.12	0.01	0.06	0.01–0.12	0.02
Depression	0.08	0.03–0.13	0.004	0.07	0.02–0.12	0.009	0.06	0.01–0.12	0.04
Hostility	0.02	− 0.04–0.09	0.51	0.02	− 0.05–0.09	0.59	− 0.01	− 0.07–0.07	0.98
Interpersonal sensitivity	0.06	0.01–0.12	0.03	0.06	0.01–0.11	0.04	0.07	0.01–0.13	0.04
Insomnia	0.06	0.01–0.11	0.02	0.06	0.01–0.11	0.03	0.05	− 0.01–0.11	0.06

Data are presented as odds ratios and 95% confidence intervals (CI) using multiple logistic regression analysis for.

Model 1: age, sex and education levels adjustments.

Model 2: age, sex, education levels, body weight categories and physical activity adjustments.

Model 3: age, sex, education levels, body weight categories, physical activity, high-density lipoprotein, fasting glucose, triglycerides and cigarette smoking adjustments.

*BSRS-5* brief symptom rating scale-5.

Table 3 shows the risk estimates of symptomatic mental stress for decayed teeth ≥ 2 and localized severer periodontitis. Those with more than two decayed teeth in the oral cavity had a higher risk of symptomatic mental distress in Model 1 [OR: 3.80 (95% CI: 1.69–8.55)], Model 2 [OR: 3.23 (95% CI: 1.37–7.64)] and Model 3 [OR: 3.59 (95% CI: 1.52–8.46)]. However, the association between localized severer periodontitis and mental stress was not significant in Model 3 [OR: 1.51 (95% CI: 0.60–3.80)] and in the other 2 models.

**Table 3:** Multivariable logistic regression analysis for decayed teeth ≥ 2 and localized severer periodontitis with symptomatic mental stress.

	Model 1			Model 2			Model 3		
	OR	95% CI	p-value	OR	95% CI	p-value	OR	95% CI	p-value
Decayed teeth number ≥ 2	3.80	1.69–8.55	0.001	3.23	1.37–7.64	0.008	3.59	1.52–8.46	0.003
Localized periodontitis	1.14	0.50–2.58	0.75	1.21	0.52–2.85	0.66	1.51	0.60–3.80	0.37

Data are presented as odds ratios and 95% confidence intervals (CI) using multiple logistic regression analysis for.

Model 1: age, sex and education levels adjustments.

Model 2: age, sex, education levels, body weight categories and physical activity adjustments.

Model 3: age, sex, education levels, body weight categories, physical activity, high-density lipoprotein, fasting glucose, triglycerides and cigarette smoking adjustments.

The results of the sensitivity tests for unmeasured confounding are shown in Supplemental Tables 1 and 2. We removed the unmeasured covariates in the multivariable linear and logistic regression analyses from the full covariate adjustment models, and the results were not changed. The estimates were larger in the model adjusting for the unmeasured covariates.

## Discussion

The main findings of the present study were that the number of decayed teeth was positively correlated with BSRS-5 scores, especially in anxiety, depression and interpersonal sensitivity after adjusting for potential covariates. More than two decayed teeth, but not localized severer periodontitis in the oral cavity, was associated with mental stress in military personnel in Taiwan.

Periodontal disease can be induced by mental stress through glucocorticoid hormone and catecholamine (epinephrine and norepinephrine) secretion and result in increased serum glucose and a suppressed immune response^34,35^. In addition, mental distress might also stimulate the sympathetic nervous system and reduce salivary flow, resulting in bacterial plaque accumulation^36^. Furthermore, mental stress would cause healthy-impairing behaviors, such as changes in dietary habits and nutrient intake, smoking, and a lower frequency of dental visits, and would contribute to adverse effects on oral health^18,37,38^. The majority of studies have demonstrated a positive relationship between mental stress or psychological factors and generalized periodontitis in middle-aged individuals and elderly individuals^11,18^. However, the association was absent for localized severer periodontitis among young adults in the present study. This was the first report to demonstrate a null association for the early phase of periodontal disease in young adults, despite that periodontal disease progressing to a generalized chronic condition and a high inflammatory status with aging may increase mental stress.

Previous studies have clearly revealed that mental stress might influence the development of carious lesions in children^39^. In contrast, in adults, there is a lack of sufficient evidence on the association between mental stress and dental caries^40^. Both the Brazilian^41^ and Swedish^42^ population-based studies revealed no correlations of mental stress with decayed teeth in middle-aged individuals. Nevertheless, occupation and race/ethnicity might have different effects on this relationship. For instance, mental stress was associated with greater levels of dental caries in adults with intellectual disabilities^43^, in soldiers participating in the War^44^, and in Japanese adults^45^. The present study further filled the gap for the different findings between children and middle- or old-aged adults in previous studies that in young adults, there was an association of decayed teeth with mental stress.

There were some limitations in this study. First, young women accounted for approximately one tenth of the military population, making it difficult to perform sex-specific analysis. Second, since the present study was a cross-sectional design, temporality and causality could not be assessed. Therefore, an association of dental caries with mental stress while no association for localized periodontitis might be only the characteristic of our study group. The military members might be too young to have periodontitis related adverse events, i.e., mental stress, but were more possibly to develop dental caries, which might be fixed till an occurrence of related symptoms, e.g., toothache (a relatively lower prevalence of filled teeth) in our military participants with mental stress. Third, although a number of covariates were adjusted, it was impossible to adjust all confounders, which might result in a bias. In contrast, this study also had some advantages. First, mental stress was measured by a self-report questionnaire, which could be objectively and quantitatively analyzed. Second, since the daily life of the military, such as diet, training, and stress source, was unified in the county, many unmeasured confounders were controlled at baseline. Third, all dental examinations were carried out by the same dentist, and the blood tests were performed in the central lab to reduce the examining bias.

## Conclusion

Our study findings suggest that decayed teeth, rather than localized severer periodontitis in the oral cavity, were a possible risk factor for mental stress in military young adults in Taiwan. These findings contradicted the association of mental stress with generalized periodontitis in middle- or old-aged adults and were consistent with the results for decayed teeth in children in prior studies.

## Supplementary Information

Supplementary Information.

## Data Availability

The datasets used and/or analysed during the current study available from the corresponding author on reasonable request.
