# Supplementary material for: Associations of decayed teeth and localized periodontitis with mental stress in young adults: CHIEF oral health study
Source: Sci Rep. 2022 Nov 9;12:19139. doi: 10.1038/s41598-022-23958-4 (PMC9646768; doi:10.1038/s41598-022-23958-4)
Supplement: Supplementary file 1 — Supplementary Information. [file 41598_2022_23958_MOESM1_ESM.docx]

Table 1 Multivariable Liner Regression Analysis for Decayed Tooth Numbers with BSRS-5 Score

|  |  | Model 1a  (All covariates) |  |  |  | Model 2a (Measured) |  |  |  | Model 3a (Unmeasured) |  |
| --- | --- | --- | --- | --- | --- | --- | --- | --- | --- | --- | --- |
|  | β | 95% CI | p-value |  | β | 95% CI | p-value |  | β | 95% CI | p-value |
| Decayed teeth |  |  |  |  |  |  |  |  |  |  |  |
| BSRS-5 scores | 0.23 | -0.02 – 0.47 | 0.07 |  | 0.26 | 0.01 – 0.50 | 0.03 |  | 0.29 | 0.04 – 0.53 | 0.02 |
| Anxiety | 0.06 | 0.01 – 0.11 | 0.02 |  | 0.07 | 0.01 – 0.12 | 0.01 |  | 0.08 | 0.02 – 0.13 | 0.006 |
| Depression | 0.06 | 0.01 – 0.11 | 0.03 |  | 0.07 | 0.01 – 0.12 | 0.01 |  | 0.08 | 0.02 – 0.13 | 0.006 |
| Hostility | 0.01 | -0.06 – 0.08 | 0.82 |  | 0.02 | -0.05 – 0.08 | 0.63 |  | 0.01 | -0.06 – 0.08 | 0.71 |
| Interpersonal sensitivity | 0.05 | -0.01 – 0.11 | 0.07 |  | 0.06 | -0.01 – 0.11 | 0.061 |  | 0.06 | -0.01 – 0.12 | 0.053 |
| Insomnia | 0.04 | -0.01 – 0.10 | 0.11 |  | 0.05 | -0.01 – 0.10 | 0.060 |  | 0.06 | 0.01 – 0.11 | 0.03 |

Data are presented as odds ratios and 95% confidence intervals (CI) using multiple logistic regression analysis for

Model 1a: age, sex, education levels, body weight categories, physical activity, cigarette smoking, systolic blood pressure, diastolic blood pressure, waist circumference, total cholesterol, high-density lipoprotein and fasting glucose adjustments.

Model 2a: age, sex, education levels, body weight categories, exercise frequency and cigarette smoking adjustments.

Model 3a: systolic blood pressure, diastolic blood pressure, waist circumference, total cholesterol, high-density lipoprotein and fasting glucose adjustments.

Abbreviations: BSRS-5, brief symptom rating scale-5

Table 2 Multivariable Logistic Regression Analysis for Decayed Teeth ≥2 and Localized Severer Periodontitis with Symptomatic Mental Stress

|  |  | Model 1a  (All covariates) |  |  |  | Model 2a  (Measured) |  |  |  | Model 3a  (Unmeasured) |  |
| --- | --- | --- | --- | --- | --- | --- | --- | --- | --- | --- | --- |
|  | OR | 95% CI | p-value |  | OR | 95% CI | p-value |  | OR | 95% CI | p-value |
| Decayed teeth number ≥2 | 3.40 | 1.33 – 8.69 | 0.01 |  | 3.34 | 1.40 – 7.98 | 0.007 |  | 3.82 | 1.69 – 8.66 | 0.001 |
| Localized periodontitis | 1.31 | 0.53 – 3.22 | 0.56 |  | 1.21 | 0.51 – 2.85 | 0.66 |  | 1.33 | 0.58 – 3.05 | 0.49 |

Data are presented as odds ratios and 95% confidence intervals (CI) using multiple logistic regression analysis for

Model 1a: age, sex, education levels, body weight categories, physical activity, cigarette smoking, systolic blood pressure, diastolic blood pressure, waist circumference, total cholesterol, high-density lipoprotein and fasting glucose adjustments.

Model 2a: age, sex, education levels, body weight categories, exercise frequency and cigarette smoking adjustments.

Model 3a: systolic blood pressure, diastolic blood pressure, waist circumference, total cholesterol, high-density lipoprotein and fasting glucose adjustments.
